# Fortifying Health Care Intellectual Property Transactions With Blockchain

**DOI:** 10.2196/44578

**Published:** 2023-08-18

**Authors:** Huan-Wei Liang, Yuan-Chia Chu, Tsung-Hsien Han

**Affiliations:** 1 Graduate Institute of Technology, Innovation & Intellectual Property Management National Chengchi University Taipei City Taiwan; 2 Technology Transfer Office Department of Medical Research Taipei Veterans General Hospital Taipei City Taiwan; 3 Department of Information Management Taipei Veterans General Hospital Taipei City Taiwan; 4 Department of Information Management National Taipei University of Nursing and Health Sciences Taipei City Taiwan; 5 Industrial Technology Investment Corporation Taipei City Taiwan

**Keywords:** intellectual property, open innovation, blockchain, appropriability regime, health care, mobile phone

## Abstract

**Background:**

Intellectual property (IP) is a substantial competitive advantage in the health care industry. However, the COVID-19 pandemic highlighted the need for open innovation and collaboration for the greater good. Despite this, the industry faces challenges with innovation owing to organizational and departmental barriers. A secure platform is necessary to facilitate IP sharing without compromising the rights of IP owners.

**Objective:**

This study proposes a blockchain-based framework to secure IP transactions in health care and bring social impact.

**Methods:**

This study reviews existing researches, publications, practical cases, firm and organization websites, and conferences related to blockchain technology, blockchain in health care, blockchain in IP management, IP pledge research, and practice of IP management blockchain. The platform architecture has 7 components: pledgers, advanced research technology (ART), IP pledge platforms, IP databases, health care research, seeking ART, and transaction condition setting. These components work together seamlessly to support the sharing and pledging of ART and knowledge, while ensuring the platform’s transparency, security, and trust.

**Results:**

The open IP pledge framework can promote technology dissemination and use, reduce research and development costs, foster collaboration, and serve the public interest. Medical organizations’ leadership and support and active participation from stakeholders are necessary for success. By leveraging blockchain technology, the platform ensures tamper-proof and transparent transactions and protects the rights of IP owners. In addition, the platform offers incentive mechanisms through pledge tokens that encourage stakeholders to share their ART and contribute to the platform.

**Conclusions:**

Overall, the proposed framework can facilitate technological innovation, tackle various challenges, and secure IP transactions. It provides a secure platform for stakeholders to share their IP without compromising their rights, promoting collaboration and progress in the health care industry. The implementation of the framework has the potential to revolutionize the industry’s approach to innovation, allowing a more open and collaborative environment driven by the greater good.

## Introduction

### Paradox of Health Care Intellectual Property Pledge

Open innovation (OI) is a widely used approach in various managerial fields and industries that facilitates the integration of knowledge inflow and outflow and research and development (R&D) [1]. Examples of its application include pharmaceutical R&D [2], blockchain-enabled value cocreation [3], and the collaborative community of mobile health in the health care industry [4]. OI encompasses both pecuniary and nonpecuniary models, which serve different purposes beyond just business operations. As Chesbrough and Bogers [5] have defined, OI refers to “a distributed innovation process based on purposively managed knowledge flows across organizational boundaries, using pecuniary and nonpecuniary mechanisms in line with the organization’s business model.”

Building on this concept, scholars have proposed open software, open patents, and open intellectual property (IP) pledge approaches where inventors or pledgers share their IPs with the public to address urgent social issues, such as the COVID-19 pandemic, mpox (monkeypox) virus, and climate change, under different licensing conditions [6-11]. Some pledgers offer their IP for free, whereas others require licensing or royalty fees below the average level [12]. To foster an innovation ecosystem based on the open spirit, pledgers often require licensees to license back derivative ART to the IP pool, including source code, algorithms, copyrighted figures, and newly granted patents.

However, the implementation of OI also brings about the paradox of openness, as it fosters the creation of inventions through the integration of internal and external R&D activities but also creates the risk of knowledge and IP leakage [13]. Therefore, although pledgers share their IP to solve certain social issues, they also face difficulties in tracking and monitoring who, how, and what IP is being used subsequently, which may unexpectedly run counter to the pledge’s goals. The markets for inventions face market failures stemming from the opportunistic assumptions of transaction cost economics (TCE) [14,15]. External organizations have high incentives to expropriate the benefits of inventions improperly without permission from IP owners or by violating license contracts signed with IP owners. The risks of controlling markets for inventions lead to several obstacles, such as difficulties in tracing IP, privatization of derivative ART, lack of robust IP protection, rising transaction costs owing to multiple pledge conditions, hindering the innovation diffusion of pledged IP, difficulties in analyzing the value and impact of pledged IP, and obscure and ambiguous IP pledge terms (Table 1). These problems severely limit the use and development of original pledged IP for other potential licensees and hinder the innovation diffusion of pledged IP through derivative ART, thereby impeding innovation diffusion [16]. Without an effective tracing and monitoring mechanism, the open spirit of the pledge strategy through IP solutions is eventually hampered, and the gap in addressing these issues remains unaddressed in existing studies.

**Table 1:** Examples of the open intellectual property (IP) pledge problem^a^.

Problems	Description
IP tracing obstructions	Pledgers face difficulty in identifying the users of the pledged IP as well as how and where they are being used. In addition, it is challenging to track what forms of derivative ART (such as source codes and patents) will be generated and contributed back to the IP pool and the pledgers. It is difficult to track and monitor the innovations and solutions to social issues that the pledgers have contributed to.
Privatization of derivative ART	“Privatized licensees,” who are also potential competitors, may choose to keep the derivative ART privately without adhering to the rules of the pledge, such as GNU General Public License. These privatized licensees may then file IP litigations against both the incumbents and the pledgers, engaging in competitive activities that reduce the value of the pledged IP and hinder their availability to others.
Lack of robust IP protection	Pledgers sharing their idea, invention, patent, copyright, creation, writing, code, algorithm, etc, on the cloud platform often lacks strong protective mechanism and may lose value. IP without appropriate protection is jeopardous to adopters and licensees because they do not truly confirm and know who owns the IP. Thus, it may increase the risks of infringement when people use the IP, and it may further lead to a decrease of cooperation.
Multipledged conditions increase the transaction cost	IP pledges may not always be completely free. Some may require minimum licensing or royalty fees, whereas others may require licensing back of derivative ART. The terms of the pledge depend on various factors such as the purpose, context, technology, area, period, and rights (such as sublicensing or sub-sublicensing rights). Negotiating with multiple stakeholders to reach an agreement can be challenging and may result in increased transaction costs, such as bargaining time and signing nondisclosure agreements.
Block the innovation diffusion of pledged IP	Some adopters and competitors use freely pledged IP to create improvements and modifications (derivative ART) that they keep as private IP without following the rules and norms of open-source software. This behavior can not only impede the original IP of the pledgers but also result in IP litigation that competes with the pledgers. Such privatization and litigation can constrain the diffusion of innovation and hinder the goal of the pledge.
Hardly analyze the value of the pledge IP and its impact	With plenty of donated pledge IPs, it becomes a challenge to evaluate their relevance and value to the pledge’s target and goal. In addition, analyzing the impact of different pledge forms and strategies is a difficult task. Owing to the lack of objective outcome analysis, pledgers face difficulties in adjusting their pledge strategy to enhance its effectiveness and efficiency.
Obscure and ambiguous IP pledge terms and scope	Some firms pledge IP with undefined and unlimited licensing periods, and their terms may be broadly explained, which may lead to disputes in the future. The unclear and ambiguous nature of the explanation and period as well as the dynamic terms of the IP pledge can create confusion and disputes between pledgers and licensees.

^a^Source: integrated and summarized based on the discussion of the IP pledge problem in this study [12,17-24].

### Blockchain as a Solution for IP Sharing

How can the open IP pledge model effectively address major health care issues? One possible solution is the use of blockchain technology to manage IP rights in a way that bridges the gap between open and closed innovation and involves multiple stakeholders [25]. Blockchain’s distributed ledger system offers not only a novel technological approach but also an opportunity to rebalance power dynamics between inventors and intermediaries, creating a new economic coordination protocol that enables business innovation. This shift from sharing information to disintermediation and ultimately to OI for social good has the potential to transform the way we approach major health care issues [26,27]. To achieve this, we propose a framework for IP pledge supported by smart contracts on the blockchain [28]. This system would allow for the tracking of licensing and sublicensing projects, IP sales, and royalty fees as well as the automatic recognition of inventor contributions. With this IP sharing mechanism, inventors would be incentivized to share their ideas while still retaining IP protection, attracting interdisciplinary experts, and facilitating agreements between licensors and licensees without the need for complex negotiation processes, thereby reducing transaction costs and time.

The remaining sections of this study are structured as follows. In the *Related Work Review* section, we will examine the historical development and essence of the IP pledge context, explore its applications during the COVID-19 pandemic, address key challenges faced by the open IP pledge model, and delve into why blockchain can be a suitable solution for effective IP management. Next, we present a comprehensive platform for IP sharing in health care, providing detailed insights into its construction, content, functional mechanisms, application context, and the tangible benefits it offers for health care OI. In the *Discussion* section, we highlight the distinctive features of the platform and elaborate on its theoretical and managerial implications. Additionally, we acknowledge any limitations of this study and provide valuable recommendations for future research in this domain.

## Related Work Review

### Overview

This section illustrates the contextual development of the open-source software (OSS) spirit and the diverse forms of evolution. Later, we show how firms and organizations are willing to pledge, donate, open, or share their IP for free or limited compensation, especially during the COVID-19 pandemic. However, pledge IP also has several problems that need to be solved, such as IP tracing obstructions, privatization of derivative ART, and lack of robust IP protection. To address these problems, we introduce blockchain as a solution.

### Introduction to OSS and IP Pledge Background

In the 1970s and 1980s, there was a trend of sharing data, source code, algorithms, and related copyrights among firms, academics, programmers, and developers to enhance the development of software and systems. As an example, Linux is a prominent free and open-source operating system that grants the freedom for anyone and any organization to use, modify, and freely redistribute all source codes at the bottom layer. In the late 1990s, the “open source” movement, which involved sharing sources, gained popularity. The Open-Source Initiative was established in 1998 to promote the OSS spirit and facilitate the collaborative development and improvement of software source code [29].

At present, the OSS movement has given rise to various innovative forms, such as Linux; FreeBSD; Android; patent pledges (such as IBM’s provision of 500 software patents for free in 2005 and Elon Musk’s announcement in 2014 not to sue anyone in good faith for using Tesla’s electric vehicle technology) [30,31]; fair, reasonable. and nondiscriminatory commitment (wherein pledgers contribute IP to incumbents to facilitate standardized technologies based on the terms “fair, reasonable and non-discriminatory”) [12]; World Intellectual Property Organization (WIPO) GREEN (a web-based exchange platform for green technology built by the WIPO to address global climate change) [32]; and blockchain communities (such as Ethereum, Hyperledger, and Bitcoin) [33]. These forms of OSS overcome resource constraints for individuals and entrepreneurs, establish technological standards or de facto standards, and enable innovation diffusion [16]. For instance, IBM’s patent pledge prompted the entry of several new software start-ups into the software market [34].

Although pledgers donate and contribute their invention, idea, data, patent, and copyright (named advanced research technology [ART]) to the public without suing adopters [35], adopters (such as software developers) need to comply with the rules of different OSS organizations. As ART is a crucial IP to the open spirit of an organization, pledgers still own the IP right when they contribute their ART [17]. Therefore, OSS organizations often enact licensing rules to guide the use of ART. For instance, OSS organizations make copyleft (which combines copyright law and licensing strategy) to ask adopters to license back their derivative ART (further inventions, improvements, and modifications) after they use the open data (eg, source code) of the community [36]. Linux enacted the General Public License (GPL or GNU; one form of copyleft) to ensure the sustainability of innovation successfully rather than being used by adopters negatively [37,38]. Individuals can also post questions and problems they encountered while using Linux on related forums such as the Linux Foundation forum [39] and Linux.org forum [40]. Some would provide useful suggestions or data to help them. However, if developers do not follow the licensing rule, they will become infringers and may be sued by pledgers [41]. Thus, pledge has been recognized as a legal issue [42].

### IP Pledge and Its Relation to COVID-19

An IP pledge can be a useful tool in addressing societal issues. In late 2019, the COVID-19 pandemic presented substantial challenges to health care systems, resulting in economic downturns, social unrest, and cybersecurity threats. However, it also created an opportunity for health care institutions to overcome their inertia and collaborate across various fields [3]; industries; individuals, such as patients, health care providers, and scholars; and organizations, such as hospitals, pharmaceutical companies, information technology firms, and research institutes, at the national level [43]. In response, some IP pledges were established. For instance, the Open COVID Pledge, created by a group of voluntary scientists, engineers, and legal experts, facilitated collaboration by enabling IT firms, pharmaceutical companies, and academics to pledge their IP to combat COVID-19 as quickly as possible.

The IP offered by Open COVID Pledge included vaccines, therapies, ventilators, testing kits, apps, and other medical devices, and adopters were not required to sign any agreement to use these IP freely. In contrast, other pledges, such as Harvard–Massachusetts Institute of Technology–Stanford (HMS), required adopters to sign licensing agreements with fair licensing fees and low-cost licensed products on an individual basis [18,44]. In addition to formal IP, some pledgers such as Medtronic also shared data and designs for medical devices, and they asked adopters to make the same commitment as the pledge to prevent competitors from blocking Medtronic’s original IP through the opportunity of the pledge [18].

Not all pledges have the same requirements [12], and they may include not only IP but also data, designs, and resources. Pledgers may allow the free use of ART during specific periods and then negotiate them later; pledge claims can be dynamic and subject to change [12,18]. Although patent pledges may increase new start-ups’ participation in the innovation ecosystem, the quality of pledged patents, such as their age and citations, may impact the innovation effect [18]. Various forms of agreements, conditions, and restrictions in pledges require effective management [45]. Therefore, further strategic management and assessment of pledges are necessary to facilitate efficient matching between pledgers and adopters [19], enabling the pledge to achieve its optimal goal [18].

### Challenges of IP Pledge

However, based on previous literature and practices, pledgers have difficulty tracking and monitoring who, how, and what ART should be used sequentially, which leads to some problems with application. We illustrate 7 examples of open IP pledge problems. First, it involves IP tracing obstructions. Pledgers do not truly know who needs and uses the pledged IP, which pledged IP should be used and licensed [46], and what forms of derivative ART (such as source codes and patents) will be and contribute back to the IP pool and pledgers. Second, it involves the privatization of derivative ART. As these incumbents (pledgers, members of the IP community, and public individuals) are potential competitors, licensees may keep the derivative ART privately without following the rules (eg, GNU GPL) of the pledge to share their modified invention [41]. One example is when companies and developers within blockchain communities privately apply for patents based on open pledges without fulfilling the obligation to license back derivative works, which can potentially hinder the progress of blockchain technology [20]. Furthermore, this behavior can diminish the value of pledged IP and impede their accessibility to others [17]. Third, the lack of strong IP protection is a concern for pledgers who share their ideas, inventions, patents, copyrights, creations, writings, codes, data, algorithms, and more on cloud platforms, as they may be susceptible to losing value. Fourth, multiple pledge conditions can increase transaction costs. Although some IP pledges are free, others may require financial conditions such as limited licensing fees based on fair, reasonable, and nondiscriminatory terms [12] and others may ask for nonfinancial conditions, such as Medtronic’s request for adopters to share their modifications and improvements based on pledged ART with the public during the COVID-19 pandemic [18]. Negotiating agreements with multiple stakeholders can be challenging because of the varying purposes, contexts, technologies, geographic areas, periods, and rights (eg, sublicensing or sub-sublicensing rights) involved in IP pledges. This difficulty can lead to increased transaction costs such as time spent bargaining and signing nondisclosure agreements. Fifth, some adopters and competitors may use freely pledged IP to create derivative works as private IP without following the norms and rules of OSS. This can impede the diffusion of innovation for pledgers, as it not only blocks their original IP but can also result in further IP litigation from competitors looking to compete with them [18]. Privatization and litigation limit innovation diffusion and impair the goal of pledges. Sixth, there is a lack of analysis on the value and impact of pledged IP. With a multitude of donated pledge IPs, it can be challenging to determine their relevance to the pledge target and goal [21]. Evaluating and analyzing the influence of different pledge forms and strategies also presents a challenge. As a result, pledgers may struggle to adjust their pledge strategy and improve its effectiveness and efficiency based on objective analysis. Seventh, firms may make IP pledges on their websites, but the terms and scope of these pledges can be unclear and ambiguous. Some may pledge their IP for a specific period, such as until the end of the COVID-19 pandemic, as announced by the World Health Organization and as was done by some members of the Open COVID Pledge [8]. Toyota, for example, pledged 24,000 patents related to vehicle electrification technologies with royalty-free licenses until 2030 to encourage the development of hybrid electric vehicle technology, with written agreements required [22,23]. Although some companies make IP pledges with specific terms and durations, others may have broad or ambiguous explanations that could lead to disputes. For instance, IBM pledged 500 patents for free use in 2005, but in 2010, it sent a warning letter to TurboHercules (founded by Microsoft), accusing it of infringing on some of the pledged patents and considering it a “competitor” [24]. The unclear scope of pledged IP is another issue, as some firms pledge undefined and unlimited licensing periods, whereas others have terms that exist in broad explanations that may lead to disputes [47]. For example, the scope of code in Linux is not always clear, particularly when it runs with proprietary codes that do not comply with the GPL clause. This makes it difficult for adopters and followers to differentiate ownership of codes and can lead to disputes. The dynamic terms and scope of a pledged IP can also increase confusion and disputes between pledgers and licensees, and potential licensees may worry about possible changes and explanations of the IP pledge contract.

On the basis of this discussion, the issues with open IP pledges can be attributed to poor management quality, leading to a decrease in the incentive for IP commercialization and low motivation for sharing among pledgers [19]. Ultimately, this impedes the open nature of pledge strategies that involve IP solutions. Table 1 provides examples of the problems associated with open IP pledges.

### Blockchain and IP Management

To address the challenges discussed above open IP pledge problem and improve IP monetization, blockchain technology has emerged as a promising and innovative solution [25]. Built on principles from TCE [48] and stakeholder theory [49], blockchain is a decentralized, peer-to-peer ledger that offers features such as anonymity, immutability, encryption, traceability, data integrity, and transparency [50,51]. By managing the interactive behaviors of stakeholders, blockchain can reduce the transaction costs associated with verifying information [52], mitigating opportunistic behaviors [48], and addressing uncertainties [25,53,54].

In a centralized or hub-and-spoke transaction model, intermediaries such as agents, coordinators, platform owners, and single authorities may hold the power to manipulate transaction activities through asymmetric information, particularly when a large volume of data flow through the platform with security risks [55]. Moreover, stakeholders such as buyers and sellers may also engage in opportunistic behaviors to extract value during deal negotiations, contract implementation, and monitoring. These behaviors increase transaction costs, particularly in a dynamic and uncertain environment.

In contrast, shared governance enabled by blockchain technology provides stakeholders with equal rights to interact, trade, and share knowledge and data using trust and consensus mechanisms [25,56]. This eliminates the need for intermediaries and reduces the complexity of managing dynamic environments and uncertain behaviors by increasing transparency and catalyzing cooperation [25,57]. The shared governance of blockchain enhances transaction security by reducing the potential for manipulation and opportunism. Furthermore, the immutable feature of the blockchain (in a permissionless blockchain, not a private blockchain) makes it difficult for miners to tamper with trading records, reducing transaction costs and enabling monitoring mechanisms to detect stakeholders’ opportunistic behaviors [58].

The research on blockchain technology is still in its early stages [59], with most previous studies focusing on financial transactions and cryptocurrencies such as Bitcoin. There are still potential areas of research in other fields and management issues [60], and only a few studies have explored the application of blockchain to IP management [25]. However, there has been increasing interest in this area, with the WIPO hosting a workshop on blockchain in 2019 [61] and holding a meeting on “Blockchain White paper for IP Ecosystems” in 2021 [62]. These discussions have explored various potential applications of blockchain technology in IP management, such as patent management, trade secret management, smart IP rights and registries, IP ownership management, time-stamping, provenance authentication, evidence of use, due diligence, traceability, certification trademarks, evidence of creation, IP enforcement, anticounterfeiting, supply chain tracking, provenance, smart contracts, digital rights management, IP rights transfer or licensing, and change of legal ownership and assignment [63-65]. Three critical objectives for IP and blockchain are to bridge the IP community and the blockchain community, explore appropriate roles for the public and private sectors, and standardize IP data [63].

Bonnet and Teuteberg [27] conducted a review of 176 publications on the management of IP using blockchain technology and identified 2 main research streams. The first focuses on the transformative impact of blockchain and distributed ledger technology on IP industries, whereas the second focuses on the impact of distributed ledger technology on IP law [27]. Denter et al [25] reviewed 52 articles on how patent management could benefit from blockchain technology using transaction cost and stakeholder theories. They proposed 3 dimensions of generation, enforcement, and exploitation applications and identified 12 propositions that suggest that blockchain is conducive to patent management. These include documenting R&D outcomes by structuring, restructuring, and standardizing efficiently; integrating multiple actors to create patents; bridging the gap between open and closed innovation more efficiently; and providing an architecture to manage nondisclosure agreements and legal matters for different stakeholders. Other benefits include reconstructing inventions, strengthening the organization’s negotiation position in legal disputes, facilitating a defensive publishing strategy, providing patent marking with product architectures to evaluate the claimed damage of infringement, changing intermediaries’ roles and services, facilitating patent licensing activity by standardized agreements, expanding licensing objectives of IP artifacts, and forming a patent pool [25]. Overall, the use of blockchain in IP management is considered suitable owing to its decentralized governance mechanism and the various benefits it provides.

The current landscape of blockchain applications in IP management includes various areas such as patents, music, images, and art [25,27,66]. These applications provide the ability to quickly register copyrights; trace the full life cycle of IP, including patent term, license, assignment, and invalidation; and detect different forms of ART (such as text and music) using artificial intelligence (AI) to find counterfeiting and infringements of copyright. In addition, blockchain can serve as a tool for enforcement [67,68] and defensive publication, which involves publishing prior ART such as new ideas and technologies to prevent others from applying for patents [61].

Some interesting examples of how IP is managed include the following. The European Union Intellectual Property Office (EUIPO) collaborated with several other IP offices in 2021 to introduce a European IP register project that uses blockchain technology to automate the registration and storage of creations and data for inventors and authors. In addition, EUIPO partnered with search services, TMview, and DesignView, to provide fast, secure, and reliable information on IP rights, such as trademark and design data [69]. EUIPO also developed a blockchain authentication platform that integrates trace systems, IP enforcement, and anticounterfeiting projects to protect IP rights [70]. These developments demonstrate how blockchain technology can facilitate cross-country coordination of the IP system and drive innovation, potentially leading to the creation of a global IP system [46,71]. Another benefit of blockchain in IP is that creators can use it to transact their creations without the need for intermediaries, thus receiving fairer compensation [27]. For example, Ujo Music developed a music transaction platform using blockchain and smart contracts, enabling musicians to trade their music creations directly to listeners without the need for third-party intermediaries and increasing their profit from the creation of music [72]. This unleashed the marketing power of music art creators and strengthened the protection of copyright by tracking music transmission and serving as evidence of infringement [73]. Another company, Questel, which was founded in 2015, provides blockchain services for IP management. Customers can upload any type of IP to their blockchain platform without size limitations and receive a cryptographic fingerprint of their IP [74]. These examples show that blockchain is helpful in making IP registration and transactions more efficient, profitable, and secure for inventors.

IP management in blockchain also involves the evolution of development and service with new technologies and professional resources. In 2017, Binded built a Bitcoin blockchain platform that allowed authors to upload their images to the platform as a registration method. Authors can receive a certificate upon completing the upload to prove their copyright [75]. Binded was later acquired by Pixsy in 2019, and several IP services were added. For example, Pixsy used AI to monitor, scan, and match uploaded images on the internet to identify potential infringement and alert authors automatically when matching similar images. Pixsy also built a copyright expert team and cooperated with international law firms to recover compensation for image infringement for authors [76,77]. Some firms use AI and big data to search for evidence of infringement, such as DeviantArt, which scans nonfungible token (NFT) marketplaces to find copyright infringement, and MarqVision, which uses AI to search for counterfeits [68].

In patents, blockchain combined with other technologies and business models can provide numerous useful and powerful tools and opportunities to attract cooperators. For example, IBM’s technology (cloud, blockchain, and Watson AI) enabled IPwe to create an IP transaction platform by using blockchain and AI in 2018. This platform allowed users to identify key patent information, analyze competitors’ and customers’ weaknesses, and leverage this information on patent transactions. It also provided a Global Patent Registry, tracked historical patent records, and offered licensing and selling services [78,79]. The platform evaluated the value of IP and recorded and executed transactions through smart contracts that ensured security and confidentiality. Recently, IPwe also planned to apply NFTs in patent transactions and tokenized patents to track every commercial activity related to a patent [79,80]. In 2022, Clarivate, a patent data set and analysis company, collaborated with IPwe to improve patent analysis and accelerate innovation [79,81].

On the basis of the abovementioned information, it appears that most blockchain platforms may be able to address issues related to tracing and reducing transaction costs for specific types of IP, but it is difficult to apply in multiple types of IP by one organization alone. Furthermore, these platforms appear to be geared toward commercial transactions rather than those related to social or public goals. Therefore, a more advanced solution is required to address issues related to the spirit of OSS.

## A Framework of IP Pledge in Health Care

There are several building blocks in the application and research of the health care industry when using blockchain technology. These include data exchange and interoperability, transaction contracts, drug supply chain management, electronic health records, and electronic medical records, which are the major applications; eHealth; remote patient monitoring, which combines the use of Internet of Things devices or sensors to obtain patient data; drug counterfeit detection; collecting data and knowledge for clinical and biomedical research; clinical trials; treatment solutions; tracing pharmaceutical products; genomics; DNA research; medicine; and medical billing and bargaining. These applications have been extensively studied and applied in the health care industry [33,82-87].

Research on the intersection of blockchain, health care, and IP pledge is still lacking. There are several gaps in the current literature. First, the research on blockchain in IP management is still in its early stages, with most studies focusing on theoretical frameworks and few empirical case studies or systematic literature reviews [25,27,88,89]. Although some scholars have argued that blockchain technology can protect IP and inventions, manage licensing contracts and royalties, facilitate the motivation for creation and inventions, and enable easy access to original IP, there is still a lack of research on how blockchain can be used in IP pledges to overcome acute crises [25,90].

Second, based on the review, it appears that there are few practical cases of blockchain in health care IP management, and most of them are on private permissioned blockchains. In addition, unlike the previous practices of blockchain in health care organizations and industries, which had clear boundaries and inertia, the COVID-19 pandemic has highlighted the urgent need for cooperation and cocreation of R&D, production, and supply chains across different organizations and even countries [3]. This presents an opportunity for the health care field to expand the use of blockchain in other areas and leverage different types of knowledge. Therefore, there is a pressing need to develop an IP pledge mechanism in health care to promote innovations and inventions that address social issues.

To address the challenges of IP management in the health care field and promote knowledge sharing, we propose a framework called the platform. This framework uses smart contracts on blockchain to enable the tracking of IP sharing and evolution, including licensing and sublicensing projects, IP sales, recording of licensing and royalty fees, and derivative ART [25]. Using this framework, we aim to reduce the information asymmetry that is often manipulated by intermediaries. The platform also automatically recognizes the contributions of inventors.

## Developmental Methodology of Platform Framework

After conducting a comprehensive review of existing research, publications, cases, firm and organization websites, and conferences related to blockchain technology, blockchain in health care, blockchain in IP management, and IP pledge fields, we identified several issues. These issues include challenges in sharing and exchanging personal information in health care, a focus on one type of IP, limited use of private permissioned blockchains in IP management, and tracking obstacles in IP pledges.

To address these issues, we designed a framework called the platform. To guide the design of the platform, we chose TCE [48], stakeholder theory [49], and the technology acceptance model (TAM) as fundamental theories [91]. These theories were selected to consider the transaction cost and efficiency of blockchain technology, the needs and interests of multiple stakeholders in health care and IP pledge, and the threshold for technology adoption in health care.

The platform consists of 4 main parts, each of which operates and coordinates based on the 3 chosen theories and previous research insights. Further details and content about the platform are provided as below *Main Architecture* section and *Content of the Platform* section.

## Main Architecture

On the basis of the trusted system of pledge IP [17], the 51% attack problem (mentioned earlier), the security risks associated with clinical and health care data (even pseudonymized data) [92], and the anonymous demand for IP transactions (where licensors and licensees are reluctant to publicly disclose their identity during the licensing process), we suggest using a public permissionless blockchain (which is open for everyone to join in reading and writing, such as Bitcoin and Ethereum) instead of private permissioned blockchains (which are controlled by a central authority such as Hyperledger) as the architecture for running the platform. Although this approach has not yet been fully implemented in IP management, previous literature [25] supports it.

Moreover, previous literature has found that permissionless blockchains are more beneficial for the open nature of copyright [68]. To improve the negotiation efficiency of IP pledges, which depends on different requirements and conditions, and to reduce the cost of monitoring IP improvements, we propose using smart contracts to automatically execute transactions and pledges. This will increase trust, collaboration, and motivation between pledgers and adopters as well as enable the tracking and storage of contracts.

## Content of the Platform

### Overview

**Figure 1:**
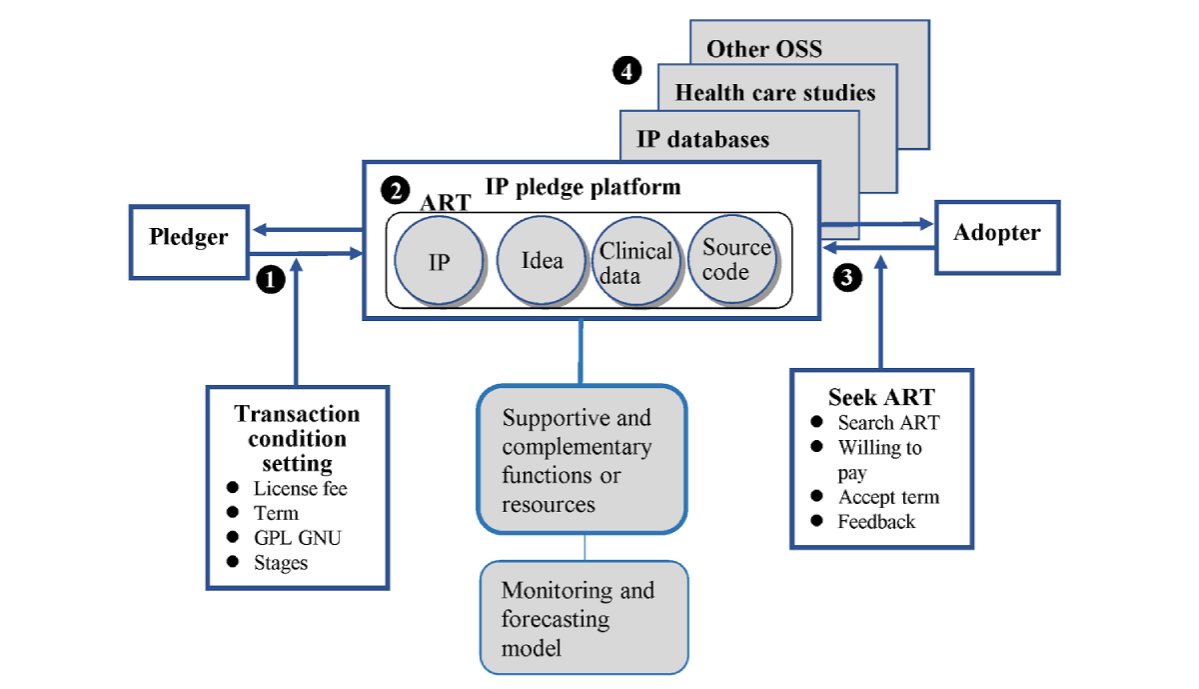
A framework of intellectual property (IP) pledge in health care. ART: advanced research technology; GNU: a recursive acronym as “GNU's Not Unix”; GPL: General Public License; OSS: open-source software.

To address large social issues efficiently and effectively through open pledge models in health care, we have developed 4 parts of the platform (as shown in Figure 1), which include uploading ART and motivation mechanisms, IP pledge and transaction platforms, ART seeking and licensing, and interoperability for interorganizations and interplatforms. This has the potential to become a global IP pledge system [71]. Each part has been designed based on insights from previous literature on blockchain, blockchain in health care, and IP management. Furthermore, each part is interconnected, operating together, and coordinating with one another. The content of the 4 parts is outlined in the following sections, including *Uploading ART and Motivation Mechanism*, *IP Pledge and Transaction Platform*, *ART Seeking and Licensing*, and *Interoperability for Interorganizations and Interplatforms* below.

### Uploading ART and Motivation Mechanism

Pledgers can comprise various entities, such as biotechnology and big pharmaceutical firms, universities, research institutions, hospitals, IT firms, individual inventors, scientists, patients, health care personnel, governments, programmers, and software engineers. They can set conditions for IP pledges, which include sharing ART based on different licensing compensation (such as free or minimum fee), terms (a period of years or an event), GPL GNU terms (freely use, license back derivative ART, or norms of pledge), and states (pledgers can set several stages to decide what extents of the content of IP or information they agree to license, trade, or disclose). Although previous research on blockchain IP management has noted the interaction of stakeholders [25] and argued for the consideration of user adoption and resistance of blockchain [60], there is little focus on how multistakeholders can adopt a specific blockchain platform.

There are several barriers that individual pledgers may face when adopting new technology and systems, such as self-efficacy barriers, traditional barriers, image barriers, threats, changes [93,94], legal impediments (such as how to draft nondisclosure agreements and licensing contracts and negotiate terms of the deal), culture of health care organizational inertia (such as high regulation, bureaucratic inefficiency, low adoption of advanced technology, and insufficient investment and human capital) [3,92], and negative attitudes (such as the findings by Hau et al [95] that medical physicians have more negative attitudes toward blockchain adoption than patients). Some solutions may be provided as a remedy; for example, user-friendly interfaces and user education have been suggested in previous studies (eg, Russo-Spena et al [3] found that training activities can help patients realize their role in the health care ecosystem and encourage them to use blockchain to participate in value cocreation) [87]. To address the abovementioned barriers and encourage IT adoption, we have used the TAM to design the platform mechanism in a way that pledgers and adopters perceive to be useful and easy to use [91]. Previous studies have also used the TAM to explain technology acceptance based on factors such as beliefs, attitudes, and intentions [96-98], and it has also been used in the health care field in the context of the COVID-19 pandemic [99].

Considering that blockchain technology can simplify the process of creating agreements and defining the terms of service, use, interacting parties, and monetization through smart contracts [28,71] as well as the fact that ART can be represented by hashed and time-stamped signals on the blockchain (on-chain) without revealing actual content (off-chain) before or during a transaction [25,100] and serve as digital certificates [68], the platform has been developed to incorporate the protection of ART into the agreement framework with secure transactions and transparent contract terms.

**Figure 2:**
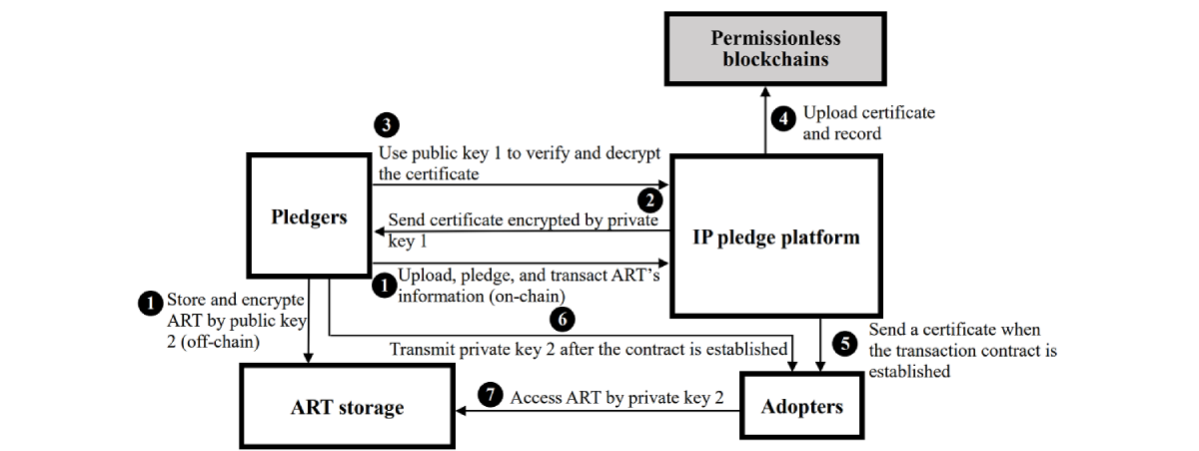
A framework of advanced research technology (ART) certificate and store. IP: intellectual property.

Ito and O’Dair [101] noted that there were discrepancies in the implementation of IP and blockchain, such as the inability of blockchain to transfer the original IP file’s size and storage effectively. Therefore, ART can be stored in off-chain storage (eg, cloud storage) and encoded with a hash into the blockchain’s on-chain to enable more efficient licensing or transfer. The mode is similar to NFT, which uses smart contracts to operate the authentication, ownership, transfer, and royalty compensation of ART in a digital form, which avoids being locked in with a particular vendor [27]. Even if pledgers only claim to open or pledge for free to use their ART rather than signing licensing contracts, they can still post the pledge claims, terms, and ART lists on the platform. The on-chain certificate and off-chain ART storage are shown in Figure 2. In the first stage (steps 1-4), pledgers upload ART on the cloud database, launch pledge, or make a deal of licensing or transaction; they send the information to the IP pledge platform. Subsequently, the platform sends a certificate encrypted by a private key to the pledgers and verifies whether the ART is uploaded to the cloud database simultaneously. Next, pledgers use a public key to verify and decrypt the certificate. The platform then uploads the certificate and records on permissionless blockchains (such as Ethereum). In the second stage (steps 5-7), while the licensing or transaction contracts of ART have been established, the platform sends a certificate to adopters. Then, pledgers send private keys to adopters who can use them to access the ART. Thus, the platform can track the upload and transaction record of the ART as well as whether each condition is achieved (eg, whether the licensing fee is paid off). Pledgers can also use the ladder disclosure of mechanism to divide specific parts of ART to decide what extent of content to disclose to adopters to overcome the problem of information paradox (ie, when licensors disclose their ART, they lose the value of the ART at the same time, especially in trade secrets) [102].

To leverage the benefits of smart contracts in the IP transaction process (eg, verifying IP authenticity, drafting agreements, tracing implementation, and payment) [71], the platform provides various conditions and contract templates and suggestions based on previous successful licensing agreements. Furthermore, it is possible to estimate the value of IP by AI, such as the Korea Technology Finance Corporation’s KIBO Patent Appraisal System, without spending plenty of money and time through experts, without needing valuation knowledge for pledgers, and without knowing specific technology and knowledge for adopters (and investors) to facilitate licensing [103]. Therefore, the platform could embed an *AI valuation system* to help pledgers understand the approximate value of ART immediately based on the information they input into it or previous successful deals. This approach reduces the technology, legal, and commerce competence gap and facilitates pledgers’ ability to set and negotiate conditions and sign contracts more easily.

To capture the interest of potential adopters, the platform offers tools that enable pledgers to create flowcharts or illustrations of their ART to clarify their concept. In addition, it provides a list of media, business, or IP law consultant firms to assist pledgers who require further guidance. Once the licensing conditions are established, pledgers can submit the information regarding their requirements to the platform. If adopters express interest in a certain ART, the Platform will notify the pledgers and match them to determine if they can accept the agreement while ensuring that the numerical content is preserved.

An effective approach to facilitate data exchange is through incentivization and funding mechanisms, as suggested in previous studies [101]. For instance, the Gene-Chain platform developed by EncryGen rewards users with “DNA tokens” for uploading their DNA profile, which can be exchanged for Bitcoin or other cryptocurrencies [3]. Another example is the blockchain platform developed by Nebula Genomics, which allows individuals to sell or rent their genome data and receive “Nebula tokens” in return. These tokens can be exchanged for a DNA report, and pharmaceutical firms and researchers can access the data to develop new drugs and conduct research [104,105].

**Figure 3:**
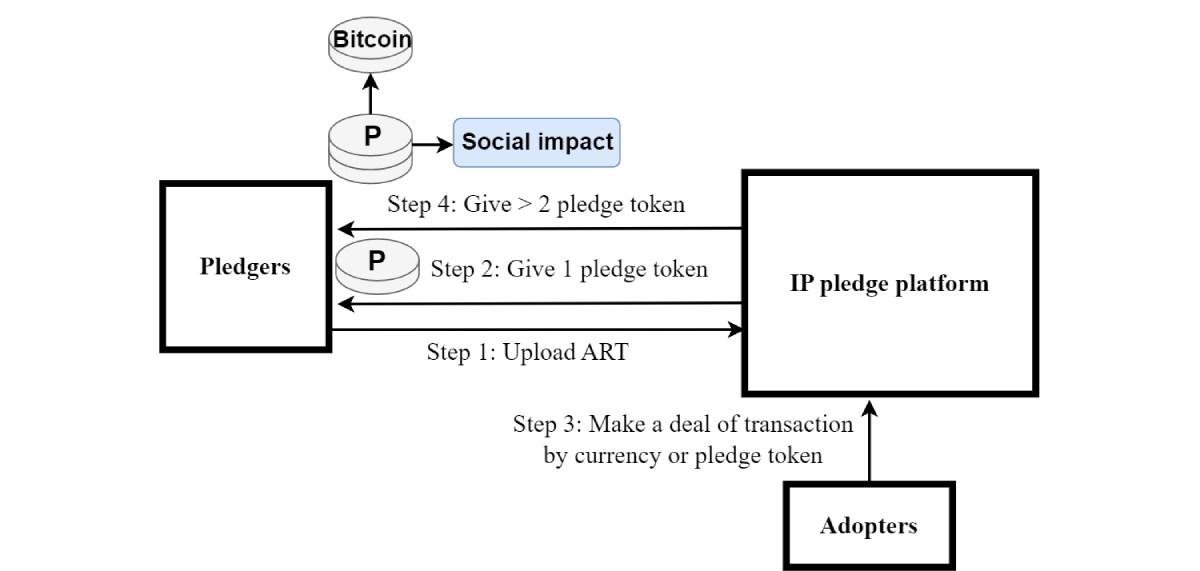
Framework of pledge token (P) and social impact. ART: advanced research technology; IP: intellectual property.

Building on the concept of incentivizing good behavior [68,106], we propose the use of “pledge tokens” to reward users who pledge their ART to the platform, thus encouraging more individuals to contribute their ART to address health care issues. When users receive “pledge tokens” upon uploading their ART, it serves as a certified claim of their IP rights, which is ensured by cryptography. Moreover, users who have successfully transacted their pledged ART can receive double “pledge tokens,” signifying their substantial social impact in addressing health care issues [106]. The “pledge tokens” can be used to exchange for other ART and cryptocurrencies, such as Bitcoin. Thus, “pledge tokens” serve as both a certified signal of contribution and a means of facilitating real IP transactions (Figure 3). We believe that such an incentive mechanism can motivate and attract more actors to participate in the value cocreation ecosystem [3].

### IP Pledge and Transaction Platform

The platform is a public permissionless blockchain that enables the recording of pledge ART, such as IP, emerging ideas, clinical data (deidentified), biomedical research data, and source code, through time-stamping with a low cost of verification [25]. These records are immutable, well protected, and easily tracked, allowing for the identification of stakeholder contributions and providing them with more incentives to share and license their ART, thereby promoting more inventive creation and cocreation [3]. Adopters such as licensees and investors can search for related ART by setting keywords, ART sorts, and conditions [68]. The platform can match pledge ART with the needs of adopters based on AI [67] and execute and track deals using smart contracts while also automatically recording licensing and royalty fee transactions. The platform can further track the derivative ART resulting from the original pledge IP and automatically transform them into a pledge IP based on the rules or norms of the OSS community. In addition, the platform can analyze the flow and impact of pledge ART, enabling pledgers to adjust their pledge policies accordingly.

The platform can be extended to connect with supportive and complementary functions or resources to enhance its effectiveness. For example, it is possible to add the monitoring and forecasting model to the platform based on public information, data, research or policy reports, and social media (eg, Facebook and Twitter) by certain prediction technology or methodology immediately, such as infodemiology approaches, which can indicate what, where, and when epidemics, infectious, illness, or disease outbreaks occur [107]. What health care, medical, and pharmaceutical products and services need the most and urgently? They are not only aware of policy makers but also health care organizations, biotechnology firms, researchers, research institutions, health care staff, and inventors to pledge their ART and devote efforts to developing needed innovation. Hence, the monitoring and forecasting model embedded in the platform could lead IP to pledge and OI activities to more specific areas, fields, groups, and targets; be aware of related stakeholders; guide, navigate, and rearrange the resources of ART; and find gaps in unmet needs.

### ART Seeking and Licensing

In their study of 32 health care technology companies using blockchain during the COVID-19 pandemic, Russo-Spena et al [3] discovered that blockchain can enable the value cocreation of data and resource sharing, patient participation, and collaboration among professionals. Furthermore, they found that blockchain facilitated 3 value cocreation activities that formed a virtuous cycle: improving service interaction, enhancing actors’ engagement, and promoting ecosystem transparency [3]. This study provides evidence that blockchain can shape the value cocreation of the health care ecosystem, encourage multistakeholder involvement, and increase opportunities for cooperation [3]. The insights gained from this study can be applied to ART pledging and transactions in health care owing to the consistent nature of codified materials, information, and data. Adopters can search for ART on the platform, indicating to what extent they are willing to compensate; which pledge conditions they accept or follow; and whether they should license improvements, derivative inventions, and IP back to pledgers and certain communities. The platform can use AI and semantic analysis to recommend appropriate ART that adopters seek based on previous successful deals in the database, thereby reducing search costs and increasing ART use [21].

### Interoperability for Interorganizations and Interplatforms

Health care data, which include patient information, drug and medical device use, clinical trials, diagnoses, research reports, and more, are highly sensitive and require security and privacy protection. Consequently, health care organizations, including hospitals, insurance companies, pharmacies, data laboratories, gene banks, and biotechnology and pharmaceutical firms, are hesitant to share data and collaborate [108]. However, blockchain technology can address this issue by creating an interoperable architecture for interorganization and platform data sharing [109]. For instance, Rana et al [108] developed a practical interoperability architecture using the Ethereum blockchain and smart contracts for multiple health care entities, including patients, physicians, chemists, and insurance companies, to exchange data while preserving the numerical content.

Smart contracts have facilitated the management of data access permissions for various health care stakeholders, with functions such as audibility, interoperability, and accessibility. For instance, Ichikawa et al [110] and their colleagues developed a mobile health system utilizing the Hyperledger Fabric blockchain network to assess the tamper resistance of cognitive-behavioral therapy data for patients living with insomnia and their health care providers. This innovative system utilizes smartphone apps to gather and secure data related to insomnia patients’ therapy progress and interactions with health care providers. By leveraging blockchain technology, the system ensures the integrity and immutability of the collected data, providing a reliable and transparent platform for both patients and health care providers to monitor and manage therapy outcomes effectively. They demonstrated the feasibility of exchanging data on a blockchain platform using smartphones, allowing users to record and license any ART (such as ideas, observations, and drawings) without location restrictions. Russo-Spena et al [3] also demonstrated the use of smartphone apps by health care technology firms to share data while controlling the target audience (such as physicians or biotechnology firms) and the duration of data sharing. In addition, WIPO has emphasized the importance of standard interoperability for connecting cross-IP databases in building a blockchain-based IP ecosystem [111]. These examples demonstrate the feasibility of exchanging data and licensing ART among health care entities using blockchain and mobile devices while preserving the numerical content.

Using the previously mentioned architecture of interoperability, a platform can be developed to connect with various IP databases, such as the United States Patent and Trademark Office and the European Patent Office, health care research institutes, and other OSS platforms. This enables other platforms to act as pledgers by donating or setting up pledge ART on the platform, which can then be matched with members of the OSS community. This approach represents the concept of a “platform of platforms,” while preserving the numerical content.

## Cost Effect and Cost Performance

The platform can improve the cost-effectiveness and cost performance of IP licensing and transactions. First, IP owners or inventors can upload the public prescription of their ART onto the platform and set their desired price. If a buyer decides to license it, the smart contract can operate automatically without incurring high negotiation and management costs, thereby reducing the transaction costs for both parties. Moreover, the anonymous mechanism provided by blockchain technology can mitigate the risk of disclosing the identities of buyers or licensees who may be secretly involved in developing crucial products in the future. Second, the platform can attract IP owners, inventors, buyers, and licensees to participate in R&D activities through a mediating mechanism without worrying about disclosing critical information. This mediating mechanism is similar to the design rule of the semiconductor industry, where integrated circuit design firms cooperate with foundries, such as the Taiwan Semiconductor Manufacturing Company, through electronic design automation without disclosing critical knowledge. They can communicate efficiently through electronic design automation rules, which have clear and specific regulations, norms, and reference guides, while preserving the numerical content and knowledge.

## Challenges and Opportunities in Health Care

The platform, which is operated by blockchain and smart contract technology, is critical in health care because of the diverse perspectives of various stakeholders, such as patients, health care professionals, developers and adopters, IP owners and buyers, policy makers, and investors. Patients are concerned about data privacy and security, whereas health care professionals prioritize proper data collection and analysis for diagnosis and study. Developers focus on the development and application of new technologies and their adherence to medical information standards. Health care providers may have reservations about the use of new technologies owing to various barriers and threats.

**Figure 4:**
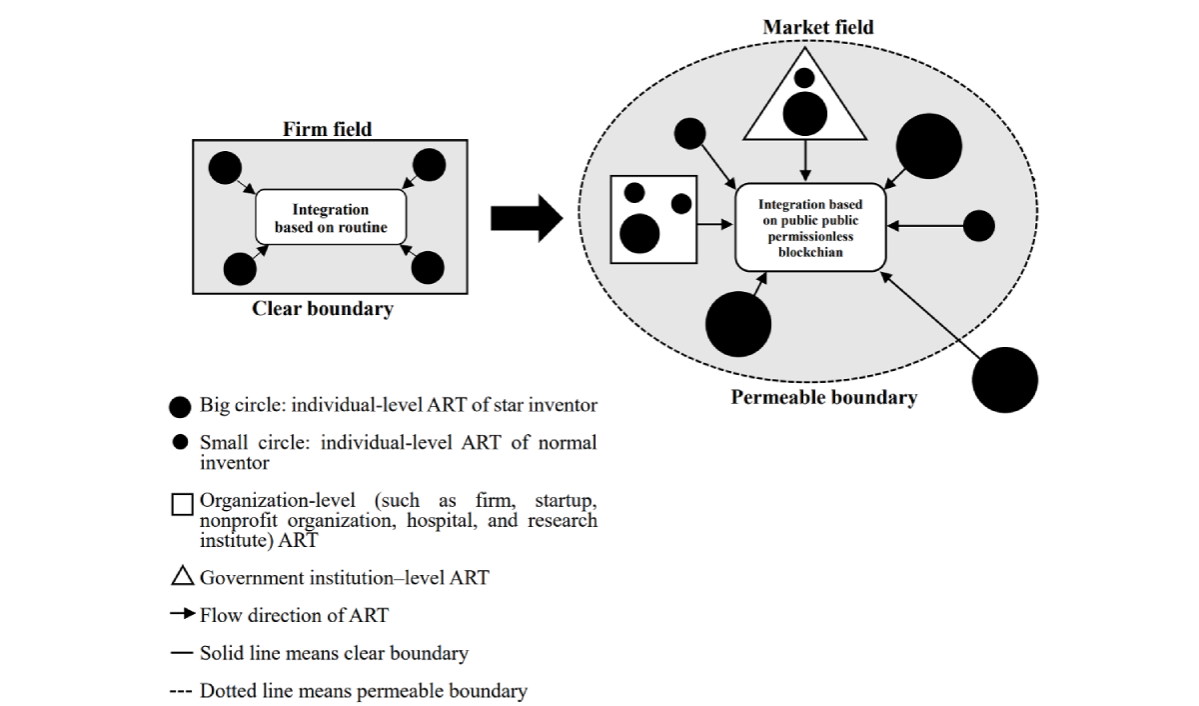
Integration of inventions from firm to market. ART: advanced research technology.

The platform addresses these challenges by integrating multi-intangible resources from various health care stakeholders through blockchain and smart contracts, which provide trust and security features. The platform also simplifies adoption through templates and suggestions that help pledgers set conditions and determine the flowchart or picture of ART to clarify the expression and address adopters’ concerns. An open and secure community is necessary to encourage stakeholders to participate in and contribute to R&D activities, thereby bridging gaps from numerous perspectives. Furthermore, the platform integrates ART from firms to markets (see Figure 4). Thus, the platform built using blockchain and smart contracts is a practical and effective solution that creates value for humanity.

## Discussion

### Overview

In this study, we review the research and practice of OSS, IP pledge challenges, and IP management blockchain and build a framework to run in the health care sector based on TCE, stakeholder theory, and the TAM. We demonstrate that knowledge to create, integrate, apply, diffuse, trace, and cocreate are crucial features and functions of the platform. We view the platform as an appropriability regime that not only helps inventors profit from their invention but also facilitates invention diffusion and integration to fill the unmet and urgent need in the health care field. In this section, we discuss the practical and managerial implications of the framework.

### Integrate Inventions From Firm to Market

The knowledge-based view holds that “knowledge” is the primary source of innovation, owned and created by individuals, and represents the most strategic resource for firms seeking to establish a competitive advantage [14] and for domestic firms to access, integrate, deploy, and construct [112]. However, the challenge that must be overcome is the transferability and integration of this knowledge [14], particularly when it is dispersed across different units, firms, and industries. As a result, Grant [14] argued that only firms can act as institutions to integrate the knowledge existing in employees and apply it to produce products more efficiently than the market. This paper presents a distinct approach using a public permissionless blockchain to create a framework for markets of inventions, ART, and data [15], which can be viewed as a market running on OSS (Figure 1). The framework functions for pledgers and adopters to offer and seek IP, ideas, clinical data (deidentified), source code, etc (ART), automatically, matching their requirements and conditions to facilitate ART transactions. Thus, we aim to extend Grant’s [14] argument to show that knowledge integration can occur in a market environment and be even more efficient than within a firm when the public is motivated to profit from innovation [113,114] and participate in activities to address social issues. The boundaries of a firm are clear, and knowledge and ART can only be integrated within it. In contrast, the boundary of a market is permeable, and knowledge and ART can be integrated across intermarkets, interdisciplinary fields, and industries. Thus, we also extend from the concept of clear organizational boundaries [1] to permeable market boundaries. Figure 4 illustrates the integration of knowledge and inventions from firms to markets. The platform is a market field that integrates diverse knowledge from stakeholders, organizations, and institutions across each boundary. This is distinct from major IP and health care blockchains, which operate within 1 organization or private community by private chain without specific issues or efficient mechanisms to facilitate and integrate knowledge flow in or out. The most critical meaning of the market field built by the platform is to shape a culture, norm, and management style to construct knowledge socially across traditional boundaries [112].

### As an Appropriability Regime to Protect IP

Maintaining OI in R&D management requires collaboration beyond a single organization. However, 2 main factors restrict the occurrence and development of OI because of the lack of a proper *appropriability regime,* which means profiting from innovation or protecting invention from imitation [115]. First, existing studies tend to focus on organizational levels and overlook individual, group, and team levels, which also have a substantial potential to spur inventions based on real-world needs. Blockchain research scholars have further suggested that the individual level is essential for innovation [60]. Second, the unintended spillover of knowledge and leakage of IP and ART pose a substantial challenge in OI collaborations, and firms often require many resources to protect their IP and ART [116]. Individuals, groups, and teams with limited resources and capabilities face even more substantial challenges in protecting their IP and ART.

Our platform addresses the problems of individual participation and unintended knowledge spillover by using blockchain and smart contract mechanisms to act as appropriability regimes [113-115] based on TCE, stakeholder theory, and the TAM. First, these mechanisms protect important ART from leakage during transactions, which can attract more individual participants to join in (Figure 2). Second, inventors can share their ART based on the gatekeeper model, which includes AI valuation, licensing conditions, smart contracts, and ladder disclosure (paying for the different disclosed extent of ART in terms of modulating and controlling the visibility and exposure of knowledge [117]). Third, the platform records all transaction processes to prevent adopters from engaging in opportunistic behaviors. Therefore, the platform, with its appropriability regime function, enables individuals, groups, teams, and other inventors to safely share their ART, reinvent, and cocreate innovations. In addition, transaction records are tamper-resistant owing to blockchain technology, which can be used as evidence for lawsuits in disputes [25] ubiquitously across industries and countries. Pledgers have the motivation to upload their ART on the platform and make a transaction or licensing agreement with a “pledge token” incentive. Thus, the trust and safety of the transaction environment facilitate each inventor’s and adopter’s contribution to the world with proper ART protection, remuneration, and interaction, rather than aggressive and improper use through unwanted behaviors. The solution of the platform addresses the abovementioned obstacles in terms of IP tracing obstructions, privatization of derivative ART, lack of robust IP protection, rising transaction costs owing to multipledged conditions, hindering the innovation diffusion of pledged IP, difficulties in analyzing the value and impact of pledged IP, and obscure and ambiguous IP pledge terms (Table 1).

A platform with a strong appropriability regime can not only enhance private returns but also gain social returns [115]. As well-protected IP is conducive to facilitating licensing and knowledge spillovers (flows), the platform can increase pledgers’ (inventors’) network access and reputational benefits [118]. Furthermore, the platform also represents *social impact* in terms of facilitating value cocreation for solving health care issues and crises [3]. Owing to the profound social impact of genuinely valuable and well-protected ART, the concept of “pledge tokens” encompasses both private and social returns. These tokens serve to facilitate the sharing and transactions of ART in a more efficient and effective manner. This is a substantial distinction from other IP and health care blockchain platforms.

### As a Solution to Address the Issue of Unbalanced Positions and Asymmetric Information

Most health care and IP blockchains operate within specific fields or items to illuminate their unique value rather than integrate cross fields or multiple items. For example, Ujo Music focuses on the copyright of music creation, Binded focuses on the copyright of image, IPwe focuses on patents, and EUIPO focuses on trademark and design data. However, these platforms were limited to transactions among firm units and certain items only, and the transaction cost was high because of the organizer’s manipulation of asymmetric information. Our platform addresses these shortcomings by allowing individuals to share their ART and by providing a trustworthy transaction environment, even in asymmetric transactions such as physicians negotiating with a firm or a small firm negotiating with a large firm.

The platform offers significant advantages for clinical researchers, including Doctors of Medicine, by providing them with valuable opportunities to integrate research and observe medical situations. They can use the platform to study patient complaints, identify service process problems, and address unmet needs for medical materials or medications [119]. This motivates Doctors of Medicine to increase their inventions and licenses at a faster pace, and the platform provides an alternative way for them to transact their inventions and IP. By lowering the threshold for ART transactions, an increasing number of people can participate, thereby stimulating greater invention production. In addition, our adoption of a public chain instead of a private one reduces the potential for improper manipulation by specific individuals or interest groups.

A public blockchain has a transparent mechanism that allows each participant to follow and trust its operations. This makes it easier to build OI ecosystems, as it eliminates the constraints of strong or weak ties among individuals, firms, and other inventive organizations [120]. In addition, it shifts the central mechanism to a decentralized one. To reduce the fear of technology and legal issues related to pledging, we used the TAM to design the platform. This has lowered the threshold of using blockchain technology and ART transactions (such as patent licensing) [91,97]. Furthermore, we extended the application of the TAM to the field of IP transactions.

The platform can disrupt the inertia and rigid boundaries of health care institutions [3] that traditionally limit certain roles (such as biotechnology companies, big pharma firms, professors, researchers, and physicians) from engaging in invention activities. It can now expand and attract new actors (such as patients, health care professionals, information technology developers, adopters from various sectors, owners, buyers of IP, policy makers, investors, nurses, and students) to participate in innovation development [43,121]. Thus, the platform could change the traditional health care invention and cooperation processes to efficient transaction, search, invention, share, and application by empowering multiple stakeholders.

### Breaking Barriers: Enabling Public Invention With Lower Thresholds

When facing institutional pressure, firms often adjust their management innovation to access external resources through OI [122]. Similarly, society needs solutions to respond to emergent and institutional pressures and the unpredictable changes and risks faced by humanity. The platform is a form of management innovation in OI that was created to address the substantial health care challenges posed by COVID-19. As societies age, health care–related issues and services become not only personal matters but also societal, national, and global concerns. The invention and innovation process are not limited to firms, but all citizens of the world have the right to contribute their talents to discoveries. Our platform is designed to achieve cost-effective and high-performance IP licensing and transactions, thereby lowering the threshold for multiple health care stakeholders to participate. This has facilitated the emergence of more innovative products and services in the market, providing patients, their families, and the public with more opportunities and choices to improve their lives.

In the post–COVID-19 period, the global society and economy gradually returned to normal life and an open model; would the value cocreation atmosphere for addressing the large health care crisis continue and develop or return to the traditional norm and inertia characteristic of health care before the pandemic outbreak as well? According to our observation in the health care field, health care policy has shifted from the flexibility of innovation (such as remote health care) for responding to COVID-19 to restricted regulation (traditional face-to-face) earlier this year. Industries face the need for organizational restructuring and increasing costs to respond to declining market demand and growing inflation, and the workforce market is confronted with volatility. For example, some high-technology firms (eg, Google, Microsoft, Meta, and Amazon) laid off >150,000 employees [123]. Thus, if health care organizations still adopt a traditional R&D approach with a high cost and inertia boundary before COVID-19 [3], it may hamper innovation after COVID-19 [124]. Firms need to plan to prepare for dealing with future pandemics or crises [125].

Our platform breaks barriers and boundaries from an individual, organization, and institution to the national level; can succeed; and further enhances the shape of the value cocreation atmosphere and norm through IP and ART transaction activities based on a secure blockchain framework. It is especially suited during the post–COVID-19 period because of the need for firm growth stemming from innovation sourced by knowledge [14,112] to overcome the volatile market. In other words, the platform serves as an efficient and effective solution for facilitating IP transactions and sharing, allowing users to easily search, apply, reinvent, and manage IP. Moreover, it presents a valuable opportunity for health care organizations to explore new avenues for reducing the risks and costs associated with innovation.

Grant and Phene [112] have specifically identified how social constructionists approach knowledge creation and its process and suggest encompassing macro institutions and individuals [14]. This study addresses these issues. By building secure and incentive mechanisms, the platform facilitates pledgers and adopters interacting with each other to construct the norm of value cocreation. By identifying what, where, and when the need and occurrence of health care issues occur, the platform navigates innovators to share their ART and put effort into addressing crises and problems immediately. The cross-border platform enhances the impact of ART and brings private and social value.

### Limitations and Future Suggestions

This study has several limitations that should be acknowledged. First, although there have been some successful cases of blockchain implementation in IP management (eg, European IP register project, Ujo Music, Binded, and IPwe), the platform itself is still only a framework for blockchain in IP pledge and has not been practically implemented. Further work is required to develop a prototype and to test its effectiveness. For instance, practitioners have noted that the high energy costs of running a blockchain need to be addressed [126]. Second, some firms have already adopted blockchain in IP management, but it still requires more analysis and effort to ensure a smooth adoption process, especially in industries with high obstacles and thresholds for adopting new technologies, such as health care [3].

Third, the “pledge tokens” that we have designed as an incentive mechanism for sharing ART and presenting social contributions to attract multiple stakeholders may require more solid support to gain the trust of potential users. For example, if the WIPO and the World Health Organization or organizations supported by public funding were to cooperate to build and operate the Platform, it would increase its legitimacy and attract more members (countries) and individuals to participate. Fourth, although open government data are promoted by many countries to encourage innovation, some barriers remain to be overcome, such as engaging diverse communities of potential users and addressing the low quality of open data [127].

Therefore, it is still unclear how organizations can be persuaded to join the Platform to share their data in a secure and interoperable manner at the initial stage. In addition, the on-chain and off-chain models for uploading ART, pledging, transacting, and storing ART may face data exchange difficulties owing to differences in storage formats. Moreover, proving the authorship of creation poses a challenge [126]. However, if a national or international organization, such as the EUIPO, with its IP register project by blockchain, participates in creating the Platform, it could help address these issues and improve efficacy and efficiency through standard formats. Future research could also explore bridging systems, such as Oracles, for off-chain and on-chain integration [68].

Finally, it should be noted that the platform serves as an institution that integrates, facilitates, and fosters the creation, exchange, application, and transaction of intangible assets, including knowledge and ART [14]. However, the problems of low quality, incorrectness, and illegality of the inputted ART have not been well addressed. Future research could focus on developing a checking mechanism or validating approach to ensure the quality and legality of the inputted ART.

The initial findings indicate that blockchain research is scattered across health care, IP management, and IP pledge. Therefore, it is worth conducting an in-depth and systematic analysis to investigate the relationships among these areas of research. As interdisciplinary research increases, it is essential to establish and operate the platform effectively and efficiently using blockchain technology for IP pledges. In addition, it is important to explore the social impact of blockchain-based IP pledges because they have the potential to unlock the contributions of multiple stakeholders from various fields and enable them to exercise their rights. Moreover, future researchers could examine the differences in the ART pledge approach in the health care sector among various countries, firms, and organizations to determine whether blockchain improves or impedes balance, equality, or OI. This exploration will allow us to observe the nature and function of blockchains in different contexts. Finally, we urge future researchers and practitioners to collaborate in the spirit of OSS to advance development and activities.

### Conclusions

Previous studies and practices of blockchain focus on a specific objective and bring us a clear and limited scope of development, whereas COVID-19 raises the storm and chaos and brings the opportunity for innovation. We have witnessed the spirit of OSS during the COVID-19 pandemic, whereas in the post–COVID-19 world, it is essential for the society to rethink how to create a positive, friendly, fair, and safe environment and mechanism to succeed in the atmosphere of value cocreation rather than close and high-cost R&D activities that exist in deep gaps between real needs and organizational inertia. The nature of the creation, search, application, and exchange of intangible assets (such as IP, knowledge, data, and ideas, which we call ART) must be aligned with sufficient support, incentives, complementary resources (eg, assets, services, technologies, knowledge, professional staff, and legal teams), and strategies to protect safely and share freely. Building on TCE, stakeholders, and TAM theories, we conclude that blockchain can be seen as having numerous roles in terms of appropriability regime, transaction platform, matchmaker, gatekeeper, catalyzer of innovation, examiner of authenticity, and prosecution of IP to integrate the works and address health care issues. It does not raise the bar; it actually lowers the threshold for different levels of stakeholders to access and share ART, especially for health care participants, and improves their influence scope based on mixed roles. Finally, we argue that even a small or seemingly insubstantial idea or invention could have a substantial and long-term impact as the building blocks of the IP pledge increase and fortify. The definitions of the key terminologies can be found in Multimedia Appendix 1 [5,12,113,115,128].

## Appendix

Multimedia Appendix 1 Definition of key terminology.

## Abbreviations

AI: artificial intelligence
ART: advanced research technology
EUIPO: European Union Intellectual Property Office
GNU: A recursive acronym as “GNU's Not Unix”
GPL: General Public License
HMS: Harvard–Massachusetts Institute of Technology–Stanford
IP: intellectual property
NFT: nonfungible token
OI: open innovation
OSS: open-source software
R&D: research and development
TAM: technology acceptance model
TCE: transaction cost economics
WIPO: World Intellectual Property Organization
