# Supplementary material for: Fortifying Health Care Intellectual Property Transactions With Blockchain
Source: J Med Internet Res. 2023 Aug 18;25:e44578. doi: 10.2196/44578 (PMC10474511; doi:10.2196/44578)
Supplement: Multimedia Appendix 1 [file jmir_v25i1e44578_app1.docx]

**Appendix**

Table 2. Definition of key terminology

| **Terminology** | **Definition** |
| --- | --- |
| Appropriability regime | A regime for profiting from innovation or protecting invention from imitation [115]. A tight appropriability regime, such as thicket patents, can enforce to exclude imitators or competitors from using the knowledge and from entering the market. The firm has specialized and cospecialized assets, such as market channels and advanced manufacturing equipment, and can occupy a good position to prevent the leakage of innovation profit and gain enough time for trials or design [113]. |
| ART | Advanced research technologies, such as inventions, emerging ideas, IP, clinical data (deidentified), biomedical research data, source code, etc. |
| FRAND | Since the owner of a standard-essential patent (SEP) has quite a power to block the development of technology in the industry, such as information communication technology, to prevent others from using SEP. Standard-setting organizations (SSOs) ask members to disclose the SEP and should license it to others on “fair, reasonable, and non-discriminatory” (FRAND) terms. That means the owner of SEP only asks for reasonable royalties rather than a maximum fee to balance the incentives of members [128]. In this study, we mean the owner of IP may pledge their IP for others to use by asking a limited royalty fee (possibly as a minimum fee) based on the spirit of FRAND. |
| GPL GNU | General Public License, a kind of licensing term in the OSS community to set the rule of IP (e.g., source code, algorithm, and patent) use, such as the licensees should share their modified invention or license back derivative art as well if they use the open source and algorithm shared in the OSS community. |
| IP | Intellectual properties consist of patents, copyright, trademark, and trade secret. It is one kind of intangible asset and the powerful competitive advantage of a firm. A firm can use IP to exclude others from entering the market or ask for compensation if occurs infringement or misappropriation. |
| OI | Open innovation is defined as “a distributed innovation process based on purposively managed knowledge flows across organizational boundaries, using pecuniary and nonpecuniary mechanisms in line with the organization’s business model. [5]” |
| Pledge | In this study, we mean the owner (licensor) of IP commit to permit others (licensee) to use their IP for free, or limited based on FRAND term so that it can accelerate innovation diffusion. Take patent as an example of pledge definition, “a patent pledge is a publicly announced intervention by patent owning entities (‘pledgers’) to out-license active patents to the restricted or unrestricted public free from or bound to certain conditions for a reasonable or no monetary compensation using standardized written or social contracts.” [12] |
